# The Atlanta Society of Medicine

**Published:** 1904-03

**Authors:** 


### *SOCIETY REPORTS.*

---

### ATLANTA SOCIETY OF MEDICINE.

Meeting of February 4. Dr. J. M. Crawford read a paper on Mastoiditis. (See page 793.)

### DISCUSSION ON DR. CRAWFORD'S PAPER.

Dr. Baird having taken the chair Dr. Stirling discussed the paper. The following is the substance of his remarks: We will omit all reference to chronic suppurating ears, to acute inflammations which show after a fair trial of all other remedies no tendency to recover, and recent acute cases with strongly marked symptoms of mastoid involvement, and confine ourselves to recent cases of acute catarrhal otitis media with some tenderness over the mastoid close behind the ear or at its tip. It is in these cases that one's treatment diverges from that of the reader of the paper. He says that the majority of his operations have been upon acute catarrhal cases. In my experience the vast majority of these cases make a good recovery with intact drums and hearing even where there is mastoid tenderness, as I believe there is in most of them, by a free opening of the drum head and even of the sagging portion of the postero-superior wall of the meatus. This encourages drainage, and combined with the other ordinary treatment usually suffices, but the opening must be large and carried well upward as well as downwards. There are many apparent reasons why this is superior to a mastoid operation, supposing it is equally efficient. Of course it is not always enough, but it generally is.

Dr. Crawford has advocated in all cases of earache, and I believe in all cases of otitis media, inflation of the middle ear with a strong blast of air. Does this account for any of his mastoid cases? What takes place under such treatment is that the germ-laden secretion in the drum is scattered in all directions, and must be driven into the mastoid cells. Of course inflation is invaluable at the proper time, but on the question of what is the proper time there is room to differ. It is one thing when the invading disease has the upper-hand, and another when it is vanquished and is retreating moribund. In the latter case Politzer bag may come in like the cavalry which the wise general hides behind a hill till the enemy has been discomfited, and is flying in disorder.

There are in relation to this disease three types of treatment, two of them I think extreme, that in which practically nothing is used except medicines, and that in which on signs of slight involvement of the mastoid an immediate mastoid operation is peiformed. The middle course is to drain as thoroughly as possible from within, and to watch with care for the development of symptoms, being prepared to operate upon the mastoid as soon as that appears advisable.

*Dr. Crawford*: Mr. Chairman, in answer to Dr. Stirling, I will say that by far the greater number of my cases of mastoiditis were brought to me after its development, and hence could not have been produced by the inflation. The case I have just reported where I operated after three days acute aural catarrh was in a case of atrophic rhinitis. In just such a case where we might look for any amount of pus, both early and late. In other cases where the conditions were more favorable the mastoiditis came on later—possibly an average time for its development was from two to four weeks after the first discharge from the ear. By a close study of these cases I have reported you will clearly see that it is the marked cases that are more dangerous. And that when we have a mastoid greatly swollen, with much pain on pressure, we have the necrosis near the outer table and that the danger of meningitis, brain abscess, etc., is much less than where we have but little pain accompanied with a little swelling. While this is a good point I must say I have never seen it presented before. Now as to the treatment of earache by inflation instead of laudanum and sweet oil poured into the ear. I feel assured I have employed this means of relief sufficiently often to form an opinion, and after careful study of these cases I can safely advise its use. The most of you gentlemen will be called upon to attend earache patients. Take with you a Politzer air-bag and inflate the ear well, if you wish to give the patient relief.

Dr. Baird presented a paper on Pneumonia. (See page 800.)

DISCUSSION DR. BAIRD'S PAPER.

*Dr. J. W. Duncan:* I was very much interested in Dr. Baird's paper, while I do not believe that the pneumoccoci are the cause of pneumonia, but only the result; yet I am afraid of them, for any germ that is capable of multiplying from a single germ in twenty-four hours to the enormous number of 16,593,673, is something to be scared at.

I would not advocate bleeding except in rare cases, when the right heart is greatly overworked in an effort to force the blood through a hepatized lung. I believe in such cases venesection might save the life of the patient, when otherwise he would die from heart failure or heart clot. I do not believe in blistering children at all, and very rarely do I advocate blistering adults. In some rare cases where resolution is very much delayed, blistering has seemed to do some good. I feel sure that I have had fine results from the application of the cotton jacket with binder and oil silk. The patients would feel more comfortable, and so express themselves. I keep the jacket on until patient is convalescent. In sthenic cases I give Norwood's tr. veratrum, combined with some opiate, as paragoric or deodorized tr. and if temperature is high I would give small doses tr. aconite, with veratrum and opiate every two hours, which will slow the action of the heart, and rest it somewhat, and thus help to prevent heart failure. I also give about two and a half grains each of camph. Dover's powder and quinine every four hours; in asthenic cases I add to same capsule capsicum and strychnia. I believe in commencing the strychnia early in the treatment, before great prostration is manifest. I believe in pneumonia we can accomplish much by appropriate use of alcoholic stimulants. As to the use of oxygen gas I have seen excellent results, especially in one desperate case in which cyanosis, delirium and picking at imaginary objects would disappear after every time the gas was inhaled, for a longer or shorter time. More than one hundred and twenty-five gallons were used in this case during three or four days.

*Dr. E. C. Cartledge:* The post-mortem findings which I have frequently seen in pneumonia have been acute nephritis and antemortem heart clot. Doesn't this suggest the free use of Lithia water and a healthy intestinal track for the kidney?

Autotoxemia from the intestinal track is likely a part of quite every febrile condition.

For the heart clot the same measures with any other aids toward lessening the hot plastic condition of the blood.

*Dr. C. W. Strickler:* I wish to call especial attention to the use of veratum viride, because it is one of the best agents we have for preventing and relieving an over-distended right heart.

---

### LEPROSY IN HAWAII.

Jas. T. Wrayson, Honolulu (*New York Medical Record,* December 19, 1903), claims that no patient should be committed to a leper colony without a bacteriologic demonstration of the disease. The method of the invasion of the organism by the bacillus of leprosy is unknown. He does not believe that the disease is hereditary, though a predisposition may be. He thinks that the bacillus has spores and that these are thrown off of the body of the leper and become lodged either in the integument or mucous membrane of a predisposed healthy person, where they remain inactive for many years, or become active within a short time. The first lesions are purely local. The activity of the spores is due to their passage through the air before entering the human body. The life of the bacillus is probably so short that it is impossible to cultivate it, hence the spores are the real danger and the foe to be combated. The author gives a history of the attempt to combat the disease by the government of Hawaii, and adds several photographs illustrating different lesions of leprosy.

---

ACCORDING to the *American Druggist* of September 28, 1903, hirudin, a styptic and antihemorrhagic for gynecological practice, is prepared by removing the heads and upper segments of leeches, extracting them with distilled water, and subsequent treatment by centrifugation and precipitation with acetic acid. It occurs as a peptone, like albumose, in brownish scales or masses, very soluble in water, but insoluble in ether or alcohol.

